# Participation of university community members in Health Promoting University (HPU) initiatives

**DOI:** 10.3389/fpubh.2023.1217177

**Published:** 2023-08-25

**Authors:** Mónica Suárez-Reyes, Stephan Van den Broucke

**Affiliations:** ^1^Escuela de Ciencias de la Actividad Fisica, Deporte y Salud, Universidad de Santiago de Chile, Santiago, Chile; ^2^Psychological Sciences Research Institute, Université Catholique de Louvain, Louvain-la-Neuve, Belgium

**Keywords:** healthy universities, health promotion, settings approach, questionnaire, participation

## Abstract

**Background:**

Several universities around the world have adopted the settings approach to health to create a Health Promoting University (HPU) initiative. Health promoting initiatives are built on the values of health promotion, with participation being one of the most important. Despite the above, there is little information on how university community members participate in HPU initiatives. This study aims to describe the participation of university community members in HPU initiatives in universities around the world.

**Methods:**

An online questionnaire was sent to representatives of universities that have implemented a HPU initiative. The questionnaire inquired about the level and nature of participation of university community members (students, professors, and administrative/technical staff) at different levels. Three levels of participation ranged from lower to higher levels were considered: (a) information delivery strategies; (b) consultation strategies and (c) involvement in design, planning and decision-making processes.

**Results:**

At least the 50% of the universities implemented strategies so that all the members of the community could participate at all levels. Information delivery strategies were the most often used, with students being the main target group. Consultation strategies were aimed mainly at students and professors, whilst professors participated most actively in the design, planning and decision-making.

**Conclusion:**

Different participation strategies are used in the HPU initiatives. Information delivery strategies, which represent the lowest level of participation, were the most often reported. Higher levels of participation were less used in the HPU initiatives. HPU initiatives should seek for strategies to provide more high-level participation to all university community members.

## 1. Introduction

Numerous universities around the world have adhered to the Health Promoting University (HPU) initiative by applying the settings approach to health (1). By assuming this approach, universities work to create an environment that promotes the health and well-being of all those who are part of its community (2). The use of the settings approach is based on the values of health promotion, of which the participation of the community is one of the most important (3).

Participation is defined as a process in which people are enabled to become actively involved in defining the issues of concern to them, in making decisions about the factors that affect their lives, and in which those involved have the opportunity to influence and take part (4, 5). It means that the actions are carried out by and with people and not on or to people (6). As the university community is made up of students, professors, and administrative/technical staff, community participation in the context of HPU is when all of these groups have the opportunity to influence and participate in the HPU initiative. As such, promoting a high level of participation by students and administrative/technical staff in the decisions that affect their learning, working and social experiences is indeed one of the 10 key characteristics that a university must have in order to be considered as health promoting (2).

Studies on community participation in other settings have described that participation can occur at different levels, taking various forms. The prototypical “ladder” model proposed by Arnstein (7) presents participation as a continuum that moves from nonparticipation (no power) via degrees of tokenism (counterfeit power) to degrees of citizen participation (actual power). At the lower levels of this continuum are the strategies in which those involved do not have any kind of power or influence, and the intervention is only an effort by those who deliver a service to correct what they consider necessary to change. At the intermediate levels are the strategies where the community receives information, can be consulted about their needs, and may have the opportunity to give an opinion, but this opinion will not necessarily be considered to change the course of the initiative. At the highest level, the community can take control and responsibility for the operation of the initiative, being truly engage and empowered.

As a classic framework that presents a spectrum of ways that stakeholders can be engaged in decisions, Arnstein’s ladder of citizen participation model has been used extensively in city planning, housing, health, and schools. In higher education, it has been used to assess the level of student engagement and participation in decision making. Despite critiques and adaptations of the model, a recent review and meta-synthesis of the literature concluded that the model has continuing value to conversations about partnership in tertiary education, along with other models (8). An alternative model to consider is that proposed by Davidson (9). This model recognizes the influence of contexts and proposes to understand participation in a non-hierarchical way, represented by a wheel distinguishing between four types of participation: information, consultation, participation and empowerment. It also allows to identify the nuances of the different types of participation. For example, information can be delivered in a minimal, limited or high quality. The same applies for other types of participation. Davidson’s model has also been used to study participation in health promotion, more specifically in promoting the active participation of local people in healthy cities initiatives (5).

Whereas participation is considered a key principle of health promotion, the evidence that links community participation to improved health status is not very strong. The reason for this is that there is no standard definition of ‘community’ and ‘participation’, hence when causal links between community participation and improved health status are found, they are situation-specific and unpredictable, and thus not generalizable (10). Nevertheless, it is generally agreed that participation may include and can lead to community uptake, ownership and sustainability for health improvements.

In universities, the level of participation by members of the university community is embedded in the university policy. Like all settings-based initiatives, HPUs consider that a high level of participation by the different members of the university community is a key element. According to the Alicante Declaration on Health Promoting Universities (11), a document created after the 8th Ibero-American HPU congress in 2018, a HPU should support the active participation of the university community in general. Special attention should thereby be given to the motivation and encouragement of students for their relevant role in both the university and social environments, and to facilitating and recognizing their active participation and real representativeness.

However, this is often difficult to achieve in practice (12, 13). This may be because many activities undertaken to promote health in the university setting only imply low levels of participation, or because not all members of the community have the same opportunities to participate at all levels. The main strategies to involve members of the university community in health actions are based on the delivery of information about health and wellbeing. For that purpose, diverse channels can be used which reach all members of the university community, but many of the activities only address health issues that concern students, leaving the rest of the university community aside (14). The same occurs with consultation strategies, which only register the needs and opinions of some groups. A high level of participation, which is essential to HPUs, is only achieved if all members of the community meaningfully participate in the design, planning or delivery of the activities (14, 15). However, this can be impeded if important decisions are in the hands of a group that concentrates all power.

The participation of the university community members in HPU initiatives, or the strategies that are used to promote participation by the university community, have not yet been extensively investigated. To our knowledge, only two studies have addressed this issue. In a study of participation in HPU initiatives, Davies and Hall (15) found that such participation may be hampered in several ways. The most obvious reasons that limit participation are the lack of resources (human and economic) and time constraints, which both are barriers to including university community members in all levels of participation. In another study, Dooris et al. (16) found that the students’ voice was often not considered when planning HPU activities. Both studies acknowledge the limited participation in HPU by members of the university community, but do not consider the strategies that are or can be used to enhance participation. Furthermore, both studies are concerned with universities in the United Kingdom, whereas the need and ways of participating in organizational processes can be very different depending on the national or cultural context.

A more extensive investigation of the participation of the university community members in HPU initiatives is therefore warranted. In this study, we investigated the participation in HPU programmes in universities around the world, and sought to explore the efforts that universities make to achieve the high levels of participation that are required for HPU initiatives.

## 2. Materials and methods

To explore the participation of the university community members, an online questionnaire survey was organized among universities belonging to HPU networks in different countries around the world. The questionnaire was an adapted version of the questionnaire used by Dooris and Doherty (17), to explore the activities carried out by healthy universities in England. In addition to various questions regarding the implementation of HPUs (18) our version of the questionnaire included questions to explore the participation of university community members. Specifically, three questions asked how students, professors, and administrative/technical staff participated in the initiative. For each of the three groups, respondents could indicate the level or type of participation in their university by choosing amongst three options representing three levels of participation that are common to Arnstein’s and Davidson’s models of community participation: (a) information delivery strategies (no power); (b) consultation strategies (counterfeit power); and (c) involvement in design, planning and decision-making (real power). Respondents could also add details in written form about the strategies of participation.

The invitation to participate in this study was sent through various HPU networks (i.e., the English network, the Ibero-American network, and several national networks). The questionnaire was made available via LimeSurvey, and had to be completed by the coordinator of the HPU or another person directly related to the initiative. The representatives of each university had to (a) be directly related to the HPU initiative in the role of coordinator, director, or assessor; and (b) have been in that position for at least 1 year before the study. Using key informants as a source of information has been used in studies in similar and other settings (17). Representatives of the universities that did not respond to the invitation sent by the network were contacted directly. At least three email reminders were sent to each potential respondent. A total of 141 universities from 48 different countries received the invitation. Of those, 54 universities from 25 countries completed the questionnaire. Most of the respondents were from Europe (*n* = 27) and the Americas (*n* = 24), with a few additional participants from Africa (*n* = 2) and Australia (*n* = 1). The universities represented in the sample were both public (*n* = 46) and private (*n* = 8) institutions. Of the completed questionnaires, 32 were answered in Spanish, 21 in English, and 1 in French.

To derive information from the data, respondents’ answers to the questionnaire were categorized according to the three levels of participation (information delivery, consultation and involvement) for the three main stakeholder groups, i.e., students, professors and administrative or technical staff. Descriptive statistics were calculated to report the number of universities that conduct information delivery strategies to students, professors, and administrative/technical staff. The same was calculated for consultation strategies and for design, planning, and decision-making. Additionally, open answers were summarized to serve as examples of strategies of participation at the different levels. The quotations serve to illustrate and reflect the words of the informants.

## 3. Results

**Figure 1:**
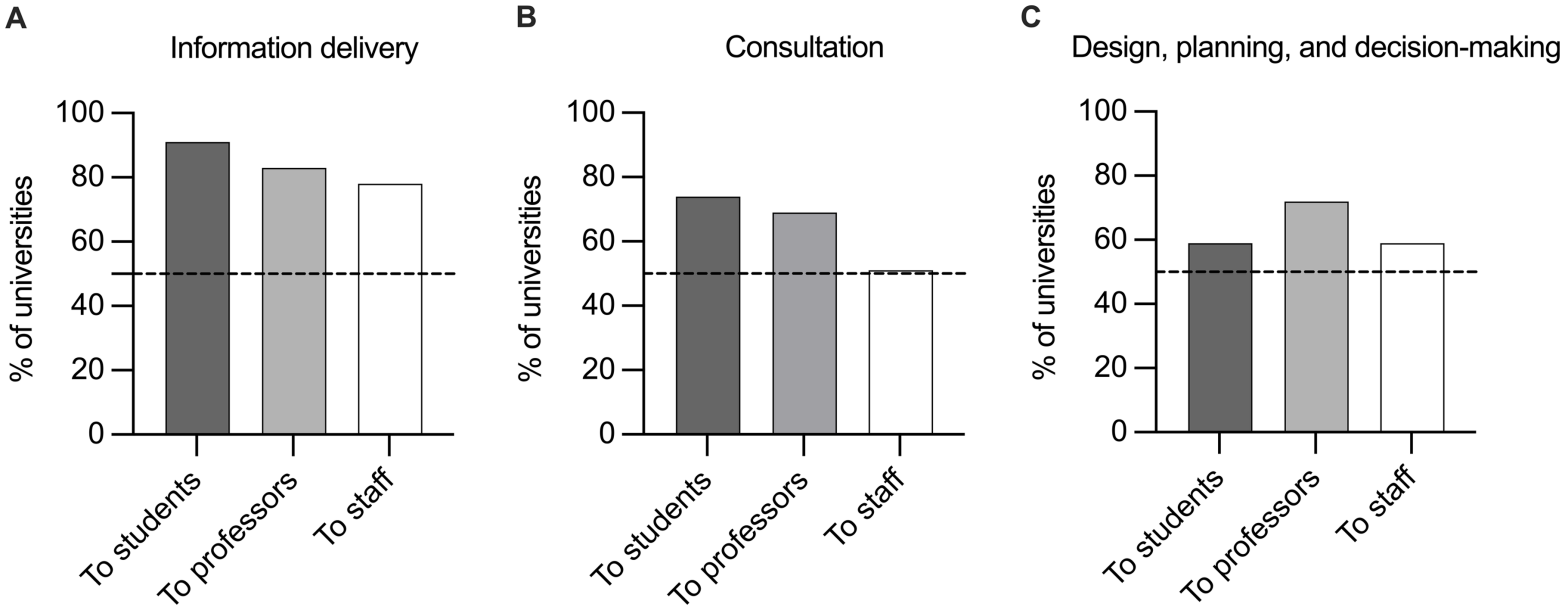
Percentage of universities addressing strategies of participation to students, professors or staff. The strategies are: **(A)** information delivery, **(B)** consultation, and **(C)** design, planning and decision-making.

Figure 1 shows the percentage of universities that implement each of the three levels of participation in relation to the three main group of stakeholders: information delivery strategies to students, professors or administrative/technical staff (A), perform consultation strategies with these groups (B), and involve them in the design, planning and decision-making of HPU initiatives (C), respectively.

Table 1 gives an overview of different HPU initiatives with the strategies that are employed to involve the three groups of stakeholders.

**Table 1:** Examples of strategies of participation of the different groups of the university community at different levels.

Information delivery strategies		
Students	Massive information delivery activities are organized by the university or external health-related organizations. Preventive health checks	Workshops Healthy fairs (with certain periodicity) Talks Conferences
	Mandatory or elective workshops with health promotion topics that are part of the academic requirements or study plan	Life skills course
	Use of technology as a communication channel	Messages through social networks Sending information through institutional email Healthy messages on the University website Healthy messages on university computer screens
Professors	Massive information delivery activities are organized by the university or external health-related organizations. Preventive health checks. Attendance to events is hampered by lack of time and academic load	Workshops Healthy fairs (with certain periodicity) Talks Conferences
Administrative/technical staff	Massive information delivery activities are organized by the university or external health-related organizations. Preventive health checks.	Workshops Healthy fairs (with certain periodicity) Talks Conferences
Consultation strategies		
Students	Surveys or questionnaires on health status and needs	Surveys on lifestyles or needs (once a year, once a semester) Risk profile as a result of surveys Participation is recognized but “partial” The surveys are organized by external organizations (ministry or health center) Participation in the definition of healthy policies
	Leaders or representatives of the student community are consulted about the needs	Student delegations (representatives, student union) student leaders
	Research	Participation in a focus group (in the framework of research) whose results can inform about the health situation and needs.
	Spontaneous and anonymous consultation methods	Other anonymous means such as consultation boards, or “clotheslines” of comments
Professors	Surveys or questionnaires on health status and needs	Risk profile as a result of surveys Surveys carried out by the occupational risk prevention service. Integral health survey
	Face-to-face consultation	Focus group (in the framework of an investigation) whose results can inform about the health situation and needs. Health talks Health reflection days Development groups by faculty
	Consultation about the learning environment	Health reflection days
Administrative/technical staff	Surveys or questionnaires on health status and needs	Surveys carried out by the occupational risk prevention service
	Face-to-face consultation	Focus group (in the framework of an investigation) whose results can inform about the health situation and needs. Health talks
Involvement in design, planning, and decision-making		
Students	Participation in health steering committees	Student health advisory committee Seed funding for health-themed research
	Design of material and information	Design of educational material Contest on healthy initiatives
	Peer educators	Self-regulation of student residences Volunteer programs Scholarships and internships with health themes
Professors	Participation in health steering committees	Professor health advisory committee Academic representation programs
	Activities design	Analysis of responses to health questionnaires Design of strategies according to specialty and experience Design and planning of activities. Evaluation of activities Seed funding with health issues Focusing lines of research on health promotion
Administrative/technical staff	Participation in health steering committees	Administrative staff health advisory committee Support in planning activities

In the next paragraphs, each of the three strategies is outlined in more detail.

### 3.1. Information delivery

When asked how they kept community members informed about health, 50 of the 54 universities mentioned that they proactively informed students; 45 also informed professors, and 42 also administrative/technical staff about health and wellbeing (Figure 1A).

The information delivery strategies mainly involved mass events (e.g., workshops, health fairs, itinerant stands, etc.), carried out inside the campus, and organized by the university or by external agencies (NGOs, Ministry of Health, etc.). The topics covered a wide range including mental health, sexual health, healthy eating, recycling strategies, etc.

“Students are invited to the development of different activities related to Health Promotion. A Health Fair is held every six months” (University in Colombia).

“Sometimes [professors] attend health fairs, respond to training programmes of recycling and storage habits” (University in Mexico).

“[The staff members] participate with the students in the events, responding to the habits that are promoted” (University in Mexico).

One university mentioned that professors’ attendance at the HPU events might be hampered due to the lack of time they have and the academic workload.

Another information delivery strategy used by some universities was to give students the possibility to attend regular courses with health promotion topics. In general, these courses were aimed only at students and were required courses to complete a curriculum. In some cases, they involved elective courses, while others were compulsory.

“Students can register for a health promotion course as one of their academic requirements” (University in Nigeria).

Aside from attending mass events and courses, the Internet was also used as a communication medium for delivering health information. Health messages were sent through Facebook or institutional mail, or placed on the university’s website.

“All are invited to participate through various media: university website, institutional mail, Facebook… we even put all kinds of healthy messages on the screens of all the computers of the university” (University in Mexico).

### 3.2. Consultation

Of the 54 universities that participated in the study, 40 indicated that they consulted students, 37 also consulted professors, and 28 also the administrative/technical staff (Figure 1B).

The most often used consultation format for all three groups was the questionnaire. These focused primarily on determining health status or exploring lifestyle habits, which were often assessed with a certain frequency (e.g., every semester or once per year). The results were used to determine the health topics that would be addressed in health promotion strategies. The questionnaires were mostly administered online, using either institutional mail or social media.

Another means of consultation used are face to face contacts with members of the three groups of the university community. In some cases, these were the official representatives (of the student bodies, professors and staff union). Students, in particular, were often interviewed for research on health and wellbeing in the university.

“[Consult student] is part of the ongoing research study, which aims to identify the key health issues to be addressed” (University in Ireland).

“They [students] are consulted with a health survey that is carried out on all newcomers to all professional careers” (University in Spain).

Some universities reported carrying out participatory diagnoses using less structured and more anonymous consultation mechanisms.

“Participatory diagnostic activities are carried out, such as "murals", where people can write their comments or experiences on the initiative” (University in Ecuador).

One university indicated that professors were not consulted about their needs or health problems, but were consulted on how the HPU initiative could support their educational goals and commitment of students. Many universities recognize that the group most consulted about their needs and health problems were the students, leaving teachers and staff out of the target of the strategies designed.

### 3.3. Involvement in design, planning and decision-making

Of the universities surveyed, 39 had mechanisms in place to involve professors in the design, planning and decision-making on health; 32 of the universities indicated that they also included students and administrative staff in this process (Figure 1C).

The participation of the university community in the design and decision-making was facilitated through various mechanisms. The most often used strategy was to incorporate of the members of the community into the health committees.

“We have a student health advisory committee who help guide our work” (University in Canada).

“Some professors are part of the steering committee… some of them are specialists in different areas addressed by the initiative for example nutrition or physical activity” (University in Spain).

For the design of strategies and activities, some universities used seed funds to which all members of the community could apply. These funds were intended to fund healthy initiatives.

Other mechanisms that were used to involve students were the creation of peer health educators or health volunteer programmes that seek to train students in various health topics so they can promote healthy lifestyles among their peers.

“[Students] can enroll in a training course for youth leaders in healthy habits” (University in Argentina).

“They [students] participate as health promoters in the form of volunteers, professional practices, social service and internships” (University in Mexico).

In some universities the activities developed by peer educators or health volunteers were carried out on campus and also in university residences.

“They lead campaigns to promote good living in university residences by carrying out self-regulation with codes of health behaviour among all inhabitants of the residences” (University in Colombia).

## 4. Discussion

The aim of this study was to document the extent to which participation is achieved in HPU initiatives, and the strategies that are used to that end. Participation is a fundamental principle that underpins health promotion. As such, it should be one of the cornerstones in health promoting initiatives that use settings approach. Like any community, the members of a university community have the right to participate in building a working and studying environment that provides good conditions for health (19). Yet despite the recognition of its importance, very little empirical information is available as to how the process of installing participation in HPU is carried out in practice (19, 20).

This study is one of the first to investigate this issue. The results show that the vast majority of universities deliver information on health to all members of the community, that most also use consultation to obtain the opinions of the different university community members, and that more than half the universities try to involve all the members of the community in the design, planning and decision-making of the health initiatives.

Among the strategies that universities use to enhance the participation of the university community in working toward a healthy university setting, information delivery to all members of the university community is the most widely used. In this way, students, teachers and staff may all benefit from the initiative. This strategy has also been used in other health-promoting settings (21). Apart from Internet, the delivery of health information through massive health events such fairs, seminars, talks and courses is the most often used channel, and is believed to generate changes in the habits of university students such as eating habits, physical activity and combatting stress (22, 23). Since young people are active users of Internet and social networking sites, this channel represents an effective resource to disseminate health messages and reach students in the university context (24, 25).

To ensure that health related activities and initiative answer the needs of the community members, consultation strategies must also be put in place. The results of this study show that consultation is often applied, with self-report surveys being the most often-used method to consult students on health issues, along with face-to-face consultations. Online surveys are a pragmatic way to get the opinion of a large group of students, but it must be kept in mind that they are not always the most appropriate method to consult people, even when the access to internet is high (6). Consultation on health through discussion and focus groups can be a good alternative. Research by Holt et al. (26) demonstrated the importance of a consultation processes, and the value for students to have the opportunity to express themselves and be listened to. It is also important that feedback is provided after the consultation, but although such feedback would signal to the members of the university community that their ideas and needs are taken into consideration, feedback after a consultation process is not common practice in HPU (6).

Participation at a high level is a key factor for the success of any health promoting initiative (11). As such, involvement in the design, planning and decision-making processes should ideally be part of a HPU initiative at every stage (6, 27). This form of participation can be promoted through different strategies, directed at all university community members including students, professors, and administrative/technical staff. Strategies that were highlighted by the participants in this survey study are the provision of seed funding for the design and planning of activities, and training community members as health promoters. The latter may enhance participants’ knowledge, skills and self-confidence, thus empowering them to become health promotion agents for their peers, friends and family (6). For students, health promotion training can be done through volunteer programmes. Volunteering facilitates the acquisition of wider life experience and is related to positive mental effects such as a higher sense of purpose, self-steem and quality of life. In addition, it facilitates the development of new job specific skill, soft skills and civic skills. Despite these advantages, students have been under-utilized and under-researched as potential volunteers in health promotion actions (28).

Previous research has shown that most HPUs develop strategies to obtain the support of the authorities of the university (29). This alignment with the authorities to develop a HPU initiative is considered as one of the most important determinants of their success (17). However, when a HPU initiative does not include the participation of all stakeholders, some groups that make up the university community may feel that the initiative is imposed on them, leading to lower acceptance (11). Promoting participation in a way that allows all members of the university community to have a voice in how the HPU initiative is conceived and implemented is an act of democracy and power sharing. However, university authorities may not want to change the existing power relations. This may be a reason why in many universities there is still a predominance of educational approaches to health promotion, rather than an approach that seeks organizational change. Questioning the power relationships within the university can be difficult to manage (30). Yet not providing members of the university community with possibilities to participate in decision-making at high levels may cause certain groups within the community to resist to the changes that a HPU initiative tries to establish (11). The results of the present study suggest that students and administrative/technical staff may be the groups that are most likely to show resistance, as they are the ones to whom universities offer less opportunities to participate at high levels.

It should be noted that there is not only a difference of status between groups of participants considered in this study, but also within these groups. Among the students, there are status differences between graduate and postgraduate students, between national and foreign students, or students in different years of study. Likewise, among academic staff there is a difference between tenured and tenure track professors or between those with full-time or part-time appointments. And among the staff there is a difference between technical staff, cleaning staff, and those who provide administrative services. Those within each group who have the least power and possibilities to participate in a HPU initiative may well be the most vulnerable to show a higher prevalence of health-adverse behaviors (30). Therefore, HPU initiatives, with the support of the authorities, should try to motivate all community members to participate, and achieve a balance between top-down commitment and bottom-up action as a way to advocate for health (31).

The study is not without limitations. A first limitation is that it only concerns a select sample of universities. Secondly, the data are based on self-reports by key informants, who answered the questions about the participation of the different groups within the university community - students, professors, and administrative/technical staff. Using key informants as a source of information has been used in other studies, but it inevitably produces a risk of bias. Moreover, the indirect way of data collection made it impossible to further refine these groups and distinguish for example between bachelor, master or PhD students, or between assistant, associate and full professors. As such, the information is concerns with larger categories that are relatively heterogeneous. It would have been useful to complement these self-reports with on-site observations and/or direct surveys with the different stakeholders, but this was not achievable within the scope and resources of the study. On the other hand, the heterogeneity of the stakeholder groups is not necessarily a weakness, as more detailed categories may have obscured larger trends. In future studies about participation in HPU, it would be interesting to analyse the differences in participation at all levels within each of these subgroups.

## 5. Conclusion

Despite these limitations, the study is one of the first to empirically document how the process of installing participation in HPU is carried out in practice. It showed that to date, the main form of participation in the HPU initiatives consists of the delivery of information. Consultation is also used, but mainly as a tool to know the health situation within the community, and to ask their opinions and needs. To make participation more meaningful, efforts should be made to ensure that consultation is accompanied by feedback. Participation of all members of the university community in the design, implementation and evaluation of HPU initiatives and in the decision-making processes is present in some of the initiatives, but needs more emphasis. To engage all those involved in HPU actions, they should not only have a voice, but also a vote (32). The type of participation toward which HPUs should direct their efforts, then, is that where members of the university community are involved in the design, implementation and evaluation of HPU initiatives and in the decision-making processes, in a meaningful way.

## Data availability statement

The raw data supporting the conclusions of this article will be made available by the authors, without undue reservation.

## Ethics statement

The studies involving humans were approved by the Ethics Committee of the Psychological Sciences Research Institute, Université catholique de Louvain, Belgium. The studies were conducted in accordance with the local legislation and institutional requirements. The participants provided their written informed consent to participate in this study.

## Author contributions

MSR was the principal investigator of the study and the major contributor to the study design, writing of the manuscript, and data analysis. SB contributed to the conception and planning of the study, supervised the data collection and analysis, and contributed to writing the manuscript. All authors have approved the final version of the manuscript and given their consent for publication.

## Funding

The study was part of a PhD research project funded through a Becas Chile scholarship to the first author (MSR).

## Conflict of interest

The authors declare that the research was conducted in the absence of any commercial or financial relationships that could be construed as a potential conflict of interest.

## Publisher’s note

All claims expressed in this article are solely those of the authors and do not necessarily represent those of their affiliated organizations, or those of the publisher, the editors and the reviewers. Any product that may be evaluated in this article, or claim that may be made by its manufacturer, is not guaranteed or endorsed by the publisher.
