# Supplementary material for: Participation of university community members in Health Promoting University (HPU) initiatives
Source: Front Public Health. 2023 Aug 25;11:1217177. doi: 10.3389/fpubh.2023.1217177 (PMC10485255; doi:10.3389/fpubh.2023.1217177)
Supplement: Supplementary file 1 [file Table_2.DOCX]

**Questionnaire on Implementation of Health Promoting Universities initiative**

Dear Participant,

We are conducting research on the implementation of Health Promoting University initiative in different contexts.

This questionnaire consists of questions about different aspects of the implementation of Health Promoting Universities. Consequently, the person who answers must be directly related to the initiative, such as the Director, Coordinator or other collaborators.

The results of this study will be used to report on the status and conditions under which Health Promoting Universities initiatives in different contexts works.

We appreciate your participation, if you want more information on the questionnaire please contact us.

**Thank you for answering this questionnaire**

**The aim of this questionnaire is to explore different aspects of the implementation process of the Health Promoting University initiative developed at your institution**

Thank you for accessing this questionnaire

**PART 1: General information**

**1.- Please provide the following information about your institution**

Please write your answer(s) here:

| Name of the institution |  |
| --- | --- |
| Country |  |
| City/Town |  |
| Post Code |  |
| Address |  |
| Email |  |
| Telephone number |  |
| Name and position of the person filling in this form |  |
| Contact information (email/telephone) |  |
| Name and position of the person in charge of the initiative (if different) |  |
| Contact information (email/telephone) |  |

**2.- Type of unit where the initiative is implemented. Please feel free to write a brief description in the space provided on the right side of each option.**

If you select 'Other', please explain your choice in the area accompanying text.

|  | Whole Institution |  |
| --- | --- | --- |
|  | Campus |  |
|  | Faculty |  |
|  | Department |  |
|  | Other |  |

**PART 2: Information about the Health Promoting University initiative**

**The following questions aim to collect information about different aspects of the Health Promoting University initiative in your institution**

**3.- What is the name of the initiative in your institution (does it have a title or ‘brand’)?**

Please write your answer here:

|  |
| --- |

**4.- When was it established? (year)**

Please write your answer here:

|  |
| --- |

**5.- Has the initiative been recognized by the authorities of the institution?**

Please choose **only one** of the following:

|  | Yes |
| --- | --- |
|  | No |

**6.- When did this recognition by the authorities of the institution occur? (year)**

**Only answer this question if the following conditions are met:**

Answer was 'Yes' at question “5” (Has the initiative been recognized by the authorities of the institution?)

Please write your answer here:

|  |
| --- |

**7.- Please provide a brief background, describing why and how the initiative was established**

Please write your answer here:

|  |
| --- |

**8.- Describe the early stages of the initiative**

Please write your answer here:

|  |
| --- |

**9.- Does the initiative have a website?**

Please choose **only one** of the following:

|  | Yes |
| --- | --- |
|  | No |

**10.- Please write the internet address URL of the initiative**

**Only answer this question if the following conditions are met:**

Answer was 'Yes' at question '9' (Does the initiative have a website?)

Please write your answer here:

|  |
| --- |

**11.- How does your institution define a “Health Promoting University”?**

Please write your answer here:

|  |
| --- |

**12.- Is your institution a member of any network of Health Promoting Universities?**

Please choose **only one** of the following:

|  | Yes |
| --- | --- |
|  | No |

**13.- Please specify to which network your institution belongs**

**Only answer this question if the following conditions are met:**

Answer was 'Yes' at question '12' (Is your institution a member of any network of Health Promoting Universities?)

Please write your answer here:

|  |
| --- |

**14.- Have the objective of the Health Promoting University initiative been established?**

Please choose **only one** of the following:

|  | Yes |
| --- | --- |
|  | No |

**15.- What are the objectives of the initiative in your institution?**

Please write your answer here:

|  |
| --- |

**PART 3: Areas of action**

The following questions aim to collect information on the **areas of action** or **strategies** developed in your institution in the framework of the Health Promoting University initiative

**16.-** **What are the areas of action or strategies that your institution has worked on or is working on?**

***Please feel free to write a brief description in the space provided on the right side of each option.***

Please choose all that apply and provide a comment:

|  | Development of healthy policies |  |
| --- | --- | --- |
|  | Creation of healthy studying environment |  |
|  | Creation of healthy working environment |  |
|  | Development of skill to improve health and wellbeing of the community members |  |
|  | Reorientation of the primary health care with focus on health promotion |  |
|  | Incorporation of health related topics in the university curriculum (regular or optional courses) |  |
|  | Incorporation of postgraduate training in health promotion |  |
|  | Support investigation in health promotion |  |
|  | Development of partnership and links with the community |  |
|  | Other |  |

**PART 4: Items of work**

The following questions aim to collect information on the **items of work** **(health topics/themes)** on which your institution works or has worked in your institution in the framework of the Health Promoting University initiative **during** **the last 5 years**

**17.- What are the items of work (health topics/themes) that your institution has worked on/is working on in the framework of the Health Promoting University initiative?**

Consider programs or plans and not occasional activities

Please feel free to write a brief description in the space provided on the right side

|  | Prevention of alcohol abuse |  |
| --- | --- | --- |
|  | Prevention of drugs abuse |  |
|  | Mental health |  |
|  | Healthy eating habits |  |
|  | Sexual health and STD/AIDS prevention |  |
|  | Road safety and transportation |  |
|  | Physical activity and sports |  |
|  | Smoking cessation |  |
|  | Promotion of free smoking spaces |  |
|  | Prevention of chronic diseases |  |
|  | Building design |  |
|  | Oral health |  |
|  | Academic performance |  |
|  | Healthy sleep |  |
|  | Inclusion of people with disabilities |  |
|  | Waste management and sustainable development |  |
|  | Safety and accident prevention |  |
|  | Other |  |

**18.-** **How were these items of work (health topics) identified or selected?***

Please write your answer here:

|  |
| --- |

* Refers to whether the items of work (health topics) were selected after a health survey, needs analysis, or other processes

**PART 4: Coordination of the initiative**

The following questions aim to collect information about how the Health Promoting University initiative is coordinated in your institution

**19.- Who leads the Health Promoting University initiative in your institution?**

***Please feel free to write a brief description in the space provided on the right side of each option***

Please choose all that apply and provide a comment:

|  | Health services |  |
| --- | --- | --- |
|  | Human resources services |  |
|  | Academic Department |  |
|  | Students’ Union |  |
|  | Other |  |

**20.- Has a senior management commitment been secured?**

Please choose **only one** of the following:

|  | Yes |
| --- | --- |
|  | No |

**21.- Please describe how the initiative obtained this senior management commitment**

**Only answer this question if the following conditions are met:**

Answer was 'Yes' at question '20' (Has a senior management commitment been secured?)

Please write your answer here:

|  |
| --- |

**22.- Please describe how the initiative plans to achieve the senior management commitment  ***

**Only answer this question if the following conditions are met:**

Answer was 'No' at question '20' (Has a senior management commitment been secured?)

Please write your answer here:

|  |
| --- |

**23.- Does the initiative have a steering/advisory group?**

Please choose **only one** of the following:

|  | Yes |
| --- | --- |
|  | No |

**24.- Does the initiative have a dedicated project coordinator/manager?**

Please choose **only one** of the following:

|  | Yes |
| --- | --- |
|  | No |

**25.- Please briefly describe details about  the project manager position, such as the grade, type and duration of the position, job description or person specification (this information will be treated confidentially).**

**Only answer this question if the following conditions are met:**

Answer was 'Yes' at question '24' (Does the initiative have a dedicated project coordinator/manager?)

Please write your answer here:

|  |
| --- |

**26.- Please describe briefly what are the key staffing resources contributing to the initiative**

Please write your answer here:

|  |
| --- |

**27.- Does your initiative have established links or partnership with external agencies or institutions?**

Please choose **only one** of the following:

|  | Yes |
| --- | --- |
|  | No |

**28.- Please describe with what kind of institutions the initiative in your institution has developed such partnership or links**

**Only answer this question if the following conditions are met:**

Answer was 'Yes' at question '27' (Does your initiative have established links or partnership with external agencies or institutions?)

Please write your answer here:

|  |
| --- |

**PART 5: Participation**

The following questions aim to collect information about the participation of community members (students, professors and administrative staff) in the development of the Health Promoting University initiative in your institution

**29.- How do students participate in the initiative of Health Promoting University?**

Please choose all that apply and provide a comment:

|  | Attendance to activities related to the initiative (lectures, workshops, fairs, etc.). |  |
| --- | --- | --- |
|  | Needs analysis where they are asked about their health problems and how to address them |  |
|  | Support, design and leadership in activities related to the initiative |  |
|  | Other |  |

**30.- How do professors participate in the initiative of Health Promoting University?**

Please choose all that apply and provide a comment:

|  | Attendance to activities related to the initiative (lectures, workshops, fairs, etc.). |  |
| --- | --- | --- |
|  | Needs analysis where they are asked about their health problems and how to address them |  |
|  | Support, design and leadership in activities related to the initiative |  |
|  | Other |  |

**31.- How do administrative staff participate in the initiative of Health Promoting University?**

Please choose all that apply and provide a comment:

|  | Attendance to activities related to the initiative (lectures, workshops, fairs, etc.). |  |
| --- | --- | --- |
|  | Needs analysis where they are asked about their health problems and how to address them |  |
|  | Support, design and leadership in activities related to the initiative |  |
|  | Other |  |

**PART 6: Evaluation**

The following questions aim to collect information about the evaluation process of the Health Promoting University initiative in your institution

**32.- Does the Health Promoting University initiative developed in your institution include an evaluation stage?**

Please choose **only one** of the following:

|  | Yes |
| --- | --- |
|  | No |

**33.- How has the institution evaluated or plans to evaluate the Health Promoting University initiative?**

**Only answer this question if the following conditions are met:**

Answer was 'Yes' at question '32' ( Does the Health Promoting University initiative developed in your institution include an evaluation stage? )

Please write your answer here:

|  |
| --- |

**PART 7: Final reflection**

**34.- If you would like to provide further information about the Health Promoting University initiative developed by your institution or a particular aspect of it, please use the space below to tell us about it in your own words**

Please write your answer here:

|  |
| --- |

**Thank you for having taken the time to complete this questionnaire and for participating in this research**
